# Retraction Note: Artificial photoactive chlorophyll conjugated vanadium carbide nanostructure for synergistic photothermal/photodynamic therapy of cancer

**DOI:** 10.1186/s12951-023-02265-8

**Published:** 2023-12-20

**Authors:** Huiting Lu, Shah Zada, Songsong Tang, Cheng Yaru, Wei Wei, Qiao Yuchun, Qiqi Yang, Jinya Du, Pengcheng Fu, Haifeng Dong, Xueji Zhang

**Affiliations:** 1 School of Chemistry and Biological Engineering, University of Science & Technology Beijing, 0 Xueyuan Road, Beijing, 100083 People’s Republic of China; 2 Marshall Laboratory of Biomedical Engineering, Research Center for Biosensor and Nanotheranostic, School of Biomedical Engineering, Shenzhen University, Guangdong, 518060 People’s Republic of China; 3 Beijing Key Laboratory for Bioengineering and Sensing Technology, Research Center for Bioengineering and Sensing Technology, School of Chemistry and Bioengineering, University of Science & Technology, Beijing, Beijing, 100083 People’s Republic of China; 4 State Key Laboratory of Marine Resource Utilization in South China, Sea Hainan University, 58 Renmin Avenue, Meilan District, Haikou, 570228 Hainan Province People’s Republic of China

**Retraction Note: Journal of Nanobiotechnology (2022) 20:121**

10.1186/s12951-022-01331-x

The Editors-in-Chief have retracted this article at the Corresponding Author’s request. After publication, concerns were raised regarding the images presented in the figures. Specifically:

Fig. 1E V2C O and N images (both showing no signal) appear to share some background noise.

Fig. 3B Chl and Chl/V2C images appear highly similar to Fig. 3F Laser and Chl/V2C (green channel only) images, respectively.

Fig. 3F Control and Chl/V2C images appear highly similar (green channel only).

Fig. 4E Chl/V2C and Laser images appear to show the same mouse; the Control tumour is at a different site compared with the other groups.

Fig. 5A:

Heart Chl/V2C and Chl/V2C + Laser 670 & 808 (nm) images appear to overlap;

Heart Laser and Chl + Laser 670 (nm) images appear to originate from the same sample;

Heart V2C + Laser 808 (nm), Chl/V2C + Laser 670 (nm) and Chl/V2C + Laser 808 (nm) images appear to originate from the same sample;

Kidney Chl/V2C and Chl/V2C + Laser 808 (nm) images appear to overlap;

Liver Chl + Laser 670 (nm) and Chl/V2C + Laser 670 & 808 (nm) images appear to overlap.

Fig. 5B TUNEL Chl/V2C and Laser images appear to overlap

Fig. 5B bottom four H&E images appear highly similar to Fig. 4A (40X) in [1].

The authors have been unable to provide the original data for validation upon request. The Editors-in-Chief therefore no longer have confidence in the presented data.

Haifeng Dong agrees to this retraction. None of the other authors have responded to any correspondence from the editor or publisher about this retraction.
